# Targeted depletion of TRBV9^+^ T cells as immunotherapy in a patient with ankylosing spondylitis

**DOI:** 10.1038/s41591-023-02613-z

**Published:** 2023-10-23

**Authors:** Olga V. Britanova, Kseniia R. Lupyr, Dmitry B. Staroverov, Irina A. Shagina, Alexey A. Aleksandrov, Yakov Y. Ustyugov, Dmitry V. Somov, Alesia Klimenko, Nadejda A. Shostak, Ivan V. Zvyagin, Alexey V. Stepanov, Ekaterina M. Merzlyak, Alexey N. Davydov, Mark Izraelson, Evgeniy S. Egorov, Ekaterina A. Bogdanova, Anna K. Vladimirova, Pavel A. Iakovlev, Denis A. Fedorenko, Roman A. Ivanov, Veronika I. Skvortsova, Sergey Lukyanov, Dmitry M. Chudakov

**Affiliations:** 1 Pirogov Russian National Research Medical University, Moscow, Russia; 2 Shemyakin and Ovchinnikov Institute of Bioorganic Chemistry, Moscow, Russia; 3 Skolkovo Institute of Science and Technology, Moscow, Russia; 4 BIOCAD, St. Petersburg, Russia; 5 Central European Institute of Technology, Masaryk University, Brno, Czech Republic; 6 MiLaboratories Inc., Sunnyvale, CA USA; 7 Department of Hematology and Chemotherapy, Pirogov National Medical and Surgical Center, Moscow, Russia; 8 Abu Dhabi Stem Cell Center, Al Muntazah, United Arab Emirates; 9 Present Address: Miltenyi Biotec B.V. & Co. KG, Bergisch Gladbach, Germany; 10 Present Address: Federal Medical Biological Agency, Moscow, Russia

**Keywords:** Adaptive immunity, Translational research, Immunotherapy

## Abstract

Autoimmunity is intrinsically driven by memory T and B cell clones inappropriately targeted at self-antigens. Selective depletion or suppression of self-reactive T cells remains a holy grail of autoimmune therapy, but disease-associated T cell receptors (TCRs) and cognate antigenic epitopes remained elusive. A TRBV9-containing CD8^+^ TCR motif was recently associated with the pathogenesis of ankylosing spondylitis, psoriatic arthritis and acute anterior uveitis, and cognate HLA-B*27-presented epitopes were identified. Following successful testing in nonhuman primate models, here we report human TRBV9^+^ T cell elimination in ankylosing spondylitis. The patient achieved remission within 3 months and ceased anti-TNF therapy after 5 years of continuous use. Complete remission has now persisted for 4 years, with three doses of anti-TRBV9 administered per year. We also observed a profound improvement in spinal mobility metrics and the Bath Ankylosing Spondylitis Metrology Index (BASMI). This represents a possibly curative therapy of an autoimmune disease via selective depletion of a TRBV-defined group of T cells. The anti-TRBV9 therapy could potentially be applicable to other HLA-B*27-associated spondyloarthropathies. Such targeted elimination of the underlying cause of the disease without systemic immunosuppression could offer a new generation of safe and efficient therapies for autoimmunity.

## Abstract (web summary)

Targeted depletion of TRBV9^+^ T cells induces remission in a single patient with ankylosing spondylitis, with significant improvements in functional and mobility metrics.

## Main

**Fig. 1:**
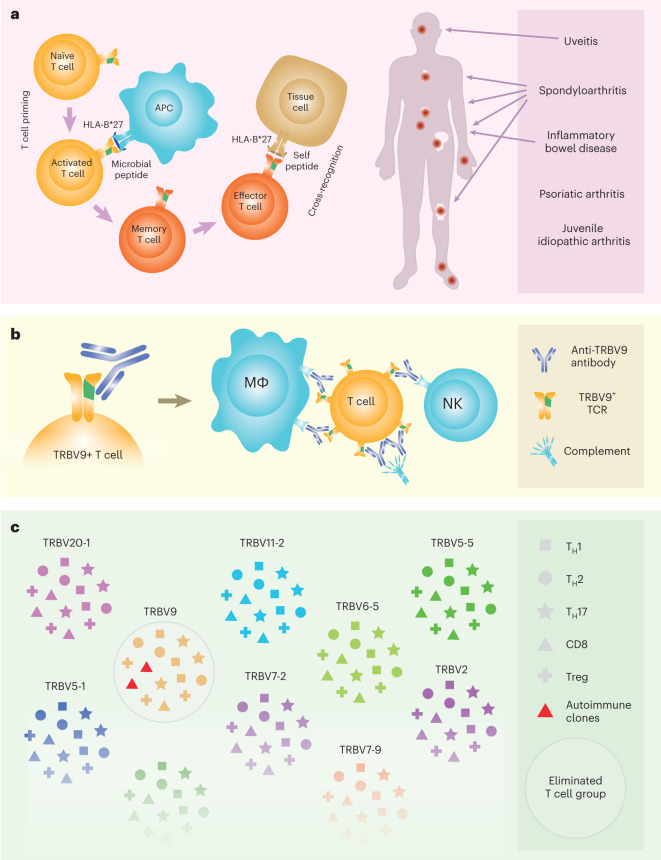
Overview of HLA-B*27-associated spondyloarthropathies, and mechanics and consequences of anti-TRBV9 immunotherapy. **a**, Concept of arthritogenic peptide. CD8^+^ T cells primed by microbial peptides presented by HLA-B*27 form memory populations that subsequently interact with HLA-B*27-bound self-peptides owing to natural cross-reactivity^22^. Depending on T cell homing and other factors, HLA-B*27-associated spondyloarthropathies manifest in a range of autoimmune diseases. **b**, Therapy with anti-TRBV9 cytotoxic antibody leads to complete elimination of TRBV9^+^ T cells via antibody-dependent cellular cytotoxicity by NK cells and complement proteins, as well as antibody-dependent cellular phagocytosis by macrophages (MΦ) such as liver Kupffer cells^23^. **c**, Anti-TRBV9 therapy eliminates TRBV9^+^ T cell clones, including autoimmune ones, but does not systemically alter any branch of T cell immunity. Only the most frequently used *TRBV* gene segments are shown.

Ankylosing spondylitis, psoriatic arthritis and other spondyloarthropathies exhibit strong association, HLA-B*27:05, suggesting a shared antigenic pathway of disease development^1^. Two groups reported a characteristic CD8^+^ T cell TCRβ CDR3 motif that is overrepresented in the peripheral blood of patients with ankylosing spondylitis compared to that of healthy HLA-B*27^+^ donors, and is also enriched in the patients’ synovial fluid compared to peripheral blood^2–4^. This motif is also expanded in the synovial tissue of HLA-B*27^+^ patients with reactive arthritis, an infection-triggered inflammatory immune response that may subsequently lead to the development of ankylosing spondylitis^5^. Furthermore, we recently described self and bacterial peptides presented by HLA-B*27:05 that are recognized by the corresponding ankylosing spondylitis-related TCRs, confirming the concept of arthritogenic peptide^1,6^. Together, the findings provide strong grounds to suggest the role of TRBV9^+^ CD8^+^ T cell clones carrying this characteristic CDR3 motif in driving autoimmunity in ankylosing spondylitis and other HLA-B*27-associated autoimmune spondyloarthropathies, including psoriatic arthritis^7^, acute anterior uveitis^6^, juvenile idiopathic arthritis and Crohn’s disease^8,9^ (Fig. 1a).

Previous studies in animal models of autoimmunity and cancer have demonstrated the efficiency and safety of therapeutic depletion of a subgroup of T cells carrying a particular TCR gene segment^10–13^.

We reasoned that the selective deletion of T cells carrying TRBV9^+^ TCRs using a cytotoxic anti-TRBV9 antibody (Fig. 1b) might provide a safe and effective therapy for HLA-B*27-associated autoimmune diseases. T cells carrying the *TRBV9* gene segment constitute about 4% of all human T cells^14^. As anti-TRBV9 therapy does not systemically suppress any branch of adaptive T cell response (such as T_H_1, T_H_2, T_H_17, T_H_1-17, Th22, Treg, T_FH_ or CD8^+^ T cells; Fig. 1c), and the remaining 96% of naive and memory TCR repertoire covers the antigenic specificities necessary for immune protection by a large margin, such therapy should not be associated with systemic immunosuppression risks.

To test this hypothesis, we first demonstrated the efficacy and safety of antibody-mediated depletion of TRBV9^+^ T cells in nonhuman primate models. Next, we performed targeted depletion of TRBV-restricted human T cells in an HLA-B*27^+^ patient with ankylosing spondylitis. This intervention resulted in profound depletion of TRBV9^+^ T cells and was followed by a dramatic improvement of disease parameters within 3 months of treatment. Some of the disease symptoms partially returned after 10 months, concomitant with the re-emergence of the pathogenic TCRβ CDR3 motif among peripheral blood T cells, thus supporting its causative role in ankylosing spondylitis. Subsequent administration of the anti-TRBV9 treatment resulted in elimination of this TCRβ CDR3 motif, followed by enduring complete remission that has persisted for 4 years to date, with a supportive regimen of anti-TRBV9 injections administered every 4 months.

With the active development of methods for identifying disease-associated TCR motifs in cognate HLA contexts^4,7,8,15–17^, we hope that in the future such targeted immunotherapeutic strategies could become applicable to at least some autoimmune pathologies.

## Preclinical studies

**Extended Data Fig. 1:**
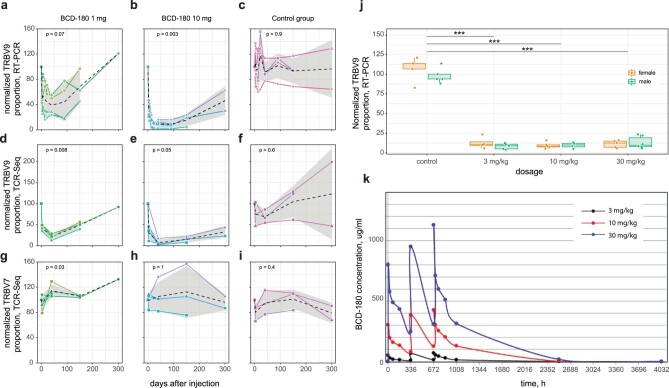
TRBV9^+^ T cell depletion in monkeys. **a-i**. TRBV9^+^ T cell depletion in *Macaca mulatta*. Plots show (a–c) Real-time PCR-based dynamic changes of *TRBV9* mRNA concentration in peripheral blood mononuclear cells (PBMCs) normalized to control *TRBV7* mRNA levels, and the proportion of (d–f) functional TRBV9^+^ and (g–i) TRBV7^+^ clonotypes within the TCRβ repertoire (normalized to the first time-point). TRBV7 data represent the sum of *TRBV7* gene segments. Kruskal-Wallis test results are shown on the top of each plot. No adjustment was made for multiple comparisons. The mean value is shown with a dotted line. Standard deviation is shown in gray. N = 4 animals per group. **j**. BCD-180-mediated TRBV9^+^ T cell depletion in *Macaca fascicularis*. Plot shows relative concentration of *TRBV9* mRNA in PBMCs 21 days after the first injection, as determined by real-time PCR and normalized to levels of *TRBV7* control mRNA. Groups were compared by type of treatment using Kruskal-Wallis test followed by Dunnett’s test. *** p < 0.001. N = 20 animals per group. The box plots show median and 1^st^–3^rd^ interquartile range. The whiskers extend from hinges to the maximum or minimum. Data beyond the 1.5 x interquartile range are plotted individually. **k**. Pharmacokinetic profile of BCD-180 in *M. fasciculari*s. Accumulation of serum BCD-180 was observed with a half-life of 9 ± 3,8 days, 12,9 ± 7 and 13,2 ± 7,2 days for 3, 10, and 30 mg kg^−^^1^ animal groups, respectively. Source data

The therapeutic-grade, cytotoxic humanized anti-TRBV9 monoclonal antibody, investigational drug BCD-180, used for the preclinical studies was produced by BIOCAD. A single intravenous (i.v.) dose of BCD-180 administered to rhesus macaques (*Macaca mulatta*) resulted in prominent depletion of peripheral blood TRBV9^+^ T cells in a dose-dependent manner, as determined by *TRBV9*-specific real-time PCR (Extended Data Fig. 1a–c) and TCR repertoire profiling (Extended Data Fig. 1d–i). In animals treated with a higher dose (10 mg i.v. per animal), this depletion lasted for ~90 days, followed by gradual reemergence of the TRBV9^+^ T cell population (Extended Data Fig. 1b). More detailed experiments were performed in *Macaca fascicularis* following i.v. administration of BCD-180 at a dose of 3, 10 or 30 mg kg^−1^ every 2 weeks for 6 weeks, with a 20-week administration-free observation period. We observed a prominent depletion of peripheral blood TRBV9^+^ T cells 21 days after administration of the first dose (Extended Data Fig. 1j). Pharmacokinetic analysis showed a typical profile for therapeutic antibodies^18^. The maximum concentration and area under the curve both increased proportionally with the dose, reflecting linear pharmacokinetics (Extended Data Fig. 1k). BCD-180 did not induce adverse effects in *M. fascicularis* or induce any local irritation at the site of administration (Supplementary Note 1).

## Case report for a patient with ankylosing spondylitis

**Fig. 2:**
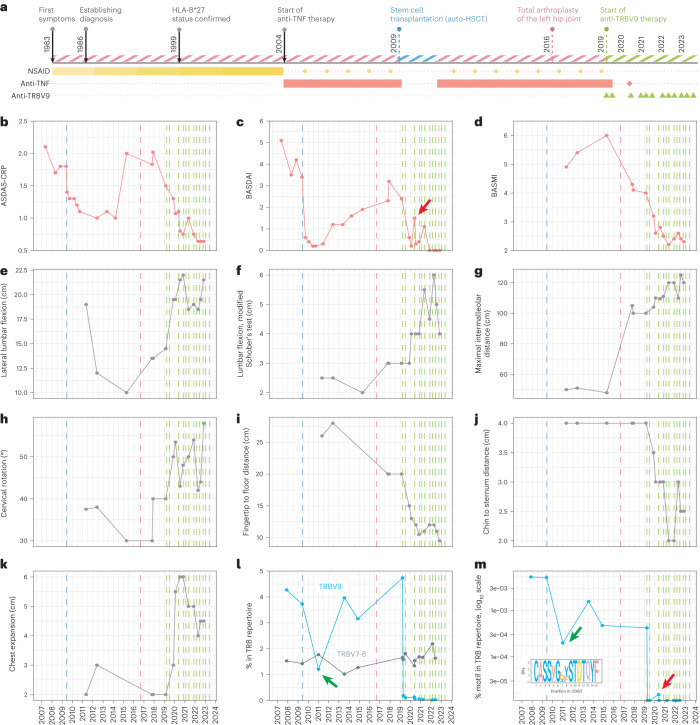
The patient’s clinical history up to and during antibody-mediated depletion of TRBV9^+^ T cells. **a**, Medical history of the patient. Pink stripes indicate periods of disease activity, and blue and green stripes show remission. Anti-TRBV9 antibody injections are indicated by green triangles. Other medications are shown as rectangles according to the period of use. Yellow gradient rectangle reflects increasing indomethacin dosage. Sporadic medications are shown with diamonds. Dashed vertical lines show autologous HSCT (blue), arthroplasty (pink) and anti-TRBV9 therapy (green). **b**, Ankylosing spondylitis disease activity score with C-reactive protein (ASDAS-CRP). **c**, BASDAI. **d**, BASMI. **e**–**k**, Spinal mobility metrics over the course of the patient’s treatment history. **l**, Proportion of TRBV9 and TRBV7-8 clonotypes in peripheral blood according to deep TCRβ repertoire profiling. **m**, Proportion of ankylosing spondylitis-associated CDR3 motif (logo shown as inset) within the total TCRβ repertoire in peripheral blood as analyzed via deep targeted *TRBV9* repertoire profiling, accounting for the proportion of TRBV9 TCRβ clonotypes in peripheral blood. Green arrows indicate depletion of TRBV9 (**l**) and ankylosing spondylitis-associated CDR3 motif (**m**) after auto-HSCT. Red arrows indicate period of relapse, which co-occurred with ankylosing spondylitis-associated CDR3 motif detection in peripheral blood. Source data

**Extended Data Fig. 2:**
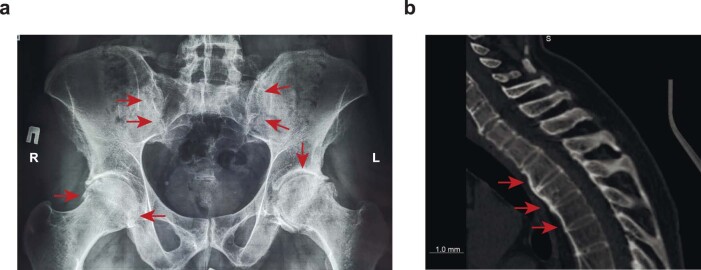
Radiographic confirmation of ankylosing spondylitis. **a**. X-ray radiography (from 2011) demonstrates complete ankylosis (grade 4 bilaterally) of sacroiliac joints. The left hip shows severe joint space narrowing, large osteophytes, severe sclerosis, and bone deformation (Kellgren-Lawrence grade 4). The right hip shows slight joint gap narrowing and a small osteophyte along the upper edge of the femoral head. **b**. Sagittal computed tomography (CT) scan of cervical thoracic part of spinal cord (2019) shows multisegmental syndesmophytes (complete anterior ossification) between Th3-Th7.

The patient, a male born in 1963, had parents with no chronic diseases and developed normally. Symptoms of ankylosing spondylitis were detected at the age of 20 after hypothermia during a hiking trip (Fig. 2a). Subsequently, a minor spinal injury was followed by progressing pathology of the spine, with general stiffness, morning pain in the lumbar spine, and pain and limited movement in the hip joints. This gradually resulted in reduced mobility of the cervical and lumbar spine. Ankylosing spondylitis was diagnosed in 1986. In 1999, an X-ray of the pelvic bones showed grade III bilateral sacroiliitis (Extended Data Fig. 2), and the patient’s HLA-B*27-positive status was confirmed. The patient underwent indomethacin therapy during the period from 1983 to 2004, with increasing dosage and diminishing efficacy, as the limited mobility gradually affected all parts of the spine. From 2004 to 2009, the patient received anti-TNF therapy, infliximab, which decreased the pain and stiffness in the spine but ceased conferring meaningful benefit after 5 years of treatment. Until 2009, disease activity remained high (Fig. 2b,c). In May 2009, the patient underwent autologous hematopoietic stem cell transplantation (HSCT)^19,20^ and entered a period of stable remission that lasted over 2 years. Notably, in this period, we observed a decreased frequency of ankylosing spondylitis-associated CDR3 motif in peripheral blood, according to both bulk TCRβ repertoire profiling and deep targeted *TRBV9* repertoire profiling (Fig. 2m, green arrow).

From 2013, disease activity had been increasing. From 2013 to 2019, the patient received anti-TNF therapy in the form of various monoclonal antibodies, including infliximab, adalimumab, certolizumab and golimumab. Each of these antibodies was effective for only 6–12 months, but after 1–2 years on alternative anti-TNF treatment(s), the patient could return to previously used anti-TNF therapies, which had relatively high efficacy once again. The patient was diagnosed with bilateral coxitis based on clinical assessment and X-ray imaging of the hip joints. In 2016, after total arthroplasty of the left hip joint, there was a significant improvement in BASMI due to an increase in the range of motion in the hip joints (Fig. 2d,g). Despite ongoing anti-TNF therapy, the patient continued to experience pain and stiffness in all parts of the spine, with severe limitation of movement in the cervical spine and pain in the hip joint.

**Extended Data Fig. 3:**
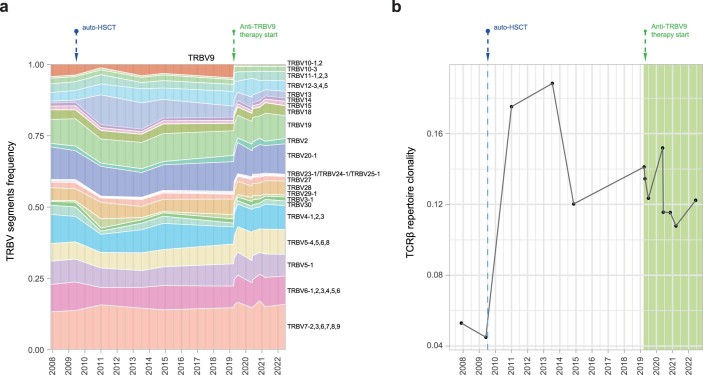
Dynamics of TRBV segments usage and TCRβ repertoire clonality during observational period. **a**. Cumulative frequency of TCRβ CDR3 clonotypes carrying different TRBV segments in the total PBMC repertoire at each time point is shown. Low frequency segments are grouped together. Note prominent decrease of TRBV9+ T cells after auto-HSCT and complete disappearance after the start of anti-TRBV9+ therapy. Note that anti-TRBV9+ therapy did not lead to depletion of any other TRBV segment. **b**. TCRβ repertoire clonality (relative presence of large clonal expansions) calculated as [1-Normalized Shannon Wiener]. Note growth of clonality after auto-HSCT, stabilization in subsequent period, and absence of prominent changes after initiation of anti-TRBV9 therapy.

**Extended Data Fig. 4:**
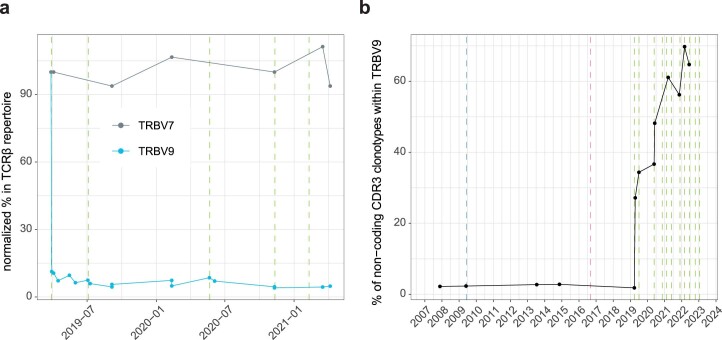
TRBV9^+^ T cell depletion. **a**. Dynamic changes relative to the first time-point for *TRBV9* and *TRBV7* mRNA concentrations in PBMCs as measured by real-time PCR and normalized to control *TRBC* mRNA. **b**. Proportion of non-functional CDR3 rearrangements within *TRBV9* repertoire. *TRBV9* repertoire profiling showed a gradual increase in the proportion of non-functional rearrangements within the remaining *TRBV9* repertoire. Such non-functional rearrangements are often present in the second copy of the TCR variable domain in mature T cells and are detectable at the level of mRNA but do not encode functional TCR chains and thus are not subjected to anti-TRBV9-mediated depletion. Considering the high proportion of non-functional mRNA within the remaining *TRBV9* repertoire after anti-TRBV9 therapy, the actual depletion of functional TRBV9+ T cells is actually ~3-fold deeper than estimated by real-time PCR in (a). Dashed vertical lines show autologous HSCT (blue), arthroplasty (pink) and anti-TRBV9 therapy (green).

The patient began treatment with the anti-TRBV9 antibody on 27 March 2019, at a dose of 60 mg i.v. The study was approved by the ethics committee of Pirogov Russian National Research Medical University (protocol no. 221). The patient provided written informed consent. Pre-treatment medication included single-dose prednisolone (120 mg i.v.), ondasetron (8 mg i.v.), chloropyramine (20 mg intramuscularly) and paracetamol (1,000 mg orally). During infusion, the patient experienced mild fatigue, nausea and transient arterial hypertension, which was most probably related to steroids. No grade 2–4 adverse events were registered. Ten days after the start of therapy, the proportion of TRBV9^+^ T cells in the peripheral blood dropped dramatically according to both bulk peripheral blood mononuclear cell (PBMC) TCRβ repertoire profiling (Fig. 2l and Extended Data Fig. 3) and real-time PCR (Extended Data Fig. 4). The ankylosing spondylitis-associated CDR3 motif disappeared from peripheral blood (Fig. 2m). At the same time, T cells carrying other TRBV segments remained unaltered (Extended Data Fig. 3a), and overall clonality of the TCRβ repertoire remained stable (Extended Data Fig. 3b).

The patient’s well-being progressively improved over 3 months after administration of the drug (Fig. 2b–k). Morning stiffness in the spine and pain in the right hip joint disappeared, and the patient’s physical activity increased. These positive effects persisted, and the patient began active exercise therapy with an emphasis on the respiratory muscles, the muscles of the anterior abdominal wall, and the limbs; such therapy was previously limited owing to severe pain in the joints and deterioration of well-being. During this period, the patient also stopped anti-TNF therapy.

To eliminate potentially remaining TRBV9^+^ T cells, a second dose (120 mg) of anti-TRBV9 therapy was administered in July 2019 although remission persisted. No side effects were observed as a result of this second, larger dose of anti-TRBV9; this was attributed to the frequency of TRBV9^+^ T cells in the blood of the patient being extremely low following the first dose (Fig. 2l).

After sustained remission, disease symptoms began to reappear in March 2020, after a period of physical over-exertion. The patient experienced pain in the upper chest, with a feeling of restriction and stiffness in the form of a ‘ring’ in the chest area. The patient also reported discomfort and a ‘feeling of heaviness’ in the lower extremities after standing for 5–10 min. On examination, clinicians noted an asymmetric tone of rectus muscles of the back, and these symptoms were reflected by an increased Bath Ankylosing Spondylitis Disease Activity Index (BASDAI) (Fig. 2c, red arrow). Remarkably, although the overall frequency of TRBV9^+^ T cells in peripheral blood remained extremely low (Fig. 2l), deep targeted profiling of the *TRBV9* TCR repertoire revealed the reappearance of ankylosing spondylitis-associated CDR3 motifs at this time (Fig. 2m, red arrow). Anti-TRBV9 therapy was therefore repeated in May 2020 (160 mg), without side effects. The symptoms disappeared completely within 10 days after injection. Notably, the ankylosing spondylitis-associated CDR3 motif also disappeared and was not observed again (Fig. 2m). Anti-TRBV9 antibody injections have been subsequently performed once every 4 months at a dose of 320–500 mg (Fig. 2a), without any detectable side effects.

At the time of writing, we have observed complete remission for 4 years since initiating anti-TRBV9 therapy based on standard disease activity indexes (Fig. 2b,c). The patient no longer receives anti-TNF therapy. During this period, we also observed improvement in spinal mobility metrics and BASMI (Fig. 2d–k). We attribute these improvements to the reduction in muscle pain and stiffness.

**Extended Data Fig. 5:**
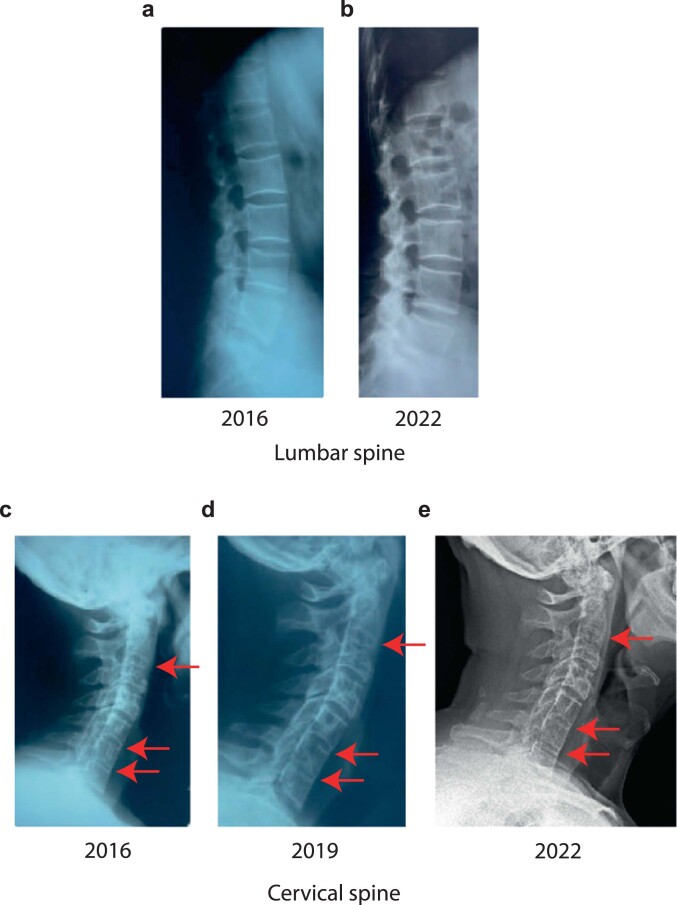
Radiography of the lumbar (a,b) and cervical (c-e) spine from 2016 to 2022. No ankylosis observed in lumbar spine, with a slight increase in the mSASSS index from 7 to 9 points. For cervical spine, ankylosis at the level of C2-C3, C5-C6, C6-C7 has been noted since 2016 (red arrows). Progression in the mSASSS index from 21 to 25 points was observed in the period 2016 to 2019, stabilized at the level of 26 points during 3 years on anti-TRBV9 therapy.

**Extended Data Fig. 6:**
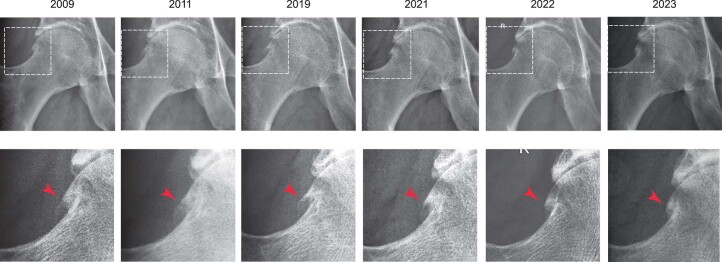
Hip X-ray images from 2009 to 2023. From 2009–2019, we observed an increase in the size of osteophytes (red arrowheads) along the upper edge of the femoral head and closure of the acetabular plate, with uneven narrowing of the joint space more along the lower edge of the head. From 2019–2023, we observed gradual degradation of the osteophyte. Lower panels show insets indicated by white dashed box in top panels.

Between 2016 and 2019, there was a radiographic progression in the cervical spine from 21 to 25 points according to the modified Stoke ankylosing spondylitis spine score (mSASSS). According to radiographic results from 2022, mSASSS stabilized at the level of 26 points (Extended Data Fig. 5b). Furthermore, X-rays of the patient’s right hip showed gradual degradation of one of the osteophytes between 2020 and 2023, which had been sequentially growing throughout the previous observation period (Extended Data Fig. 6). Evaluation of syndesmophytes, enthesophytes and other osteophytes showed no disease progression.

Here we describe targeted immunotherapy for an autoimmune disease based on a monoclonal antibody that selectively depletes a narrow TRBV-defined subgroup of T lymphocytes, which includes clones associated with the development of the autoimmunity. This intervention was successful and led to a long-term complete remission of symptoms that were not responding to existing therapeutic interventions, showing feasibility, tolerability and efficacy of antibody-mediated TRBV9^+^ T cell depletion for the treatment of ankylosing spondylitis.

The anti-TRBV9 therapy is currently in a phase II trial for ankylosing spondylitis (https://clinicaltrials.gov/ct2/show/NCT05445076) and could be applicable to other HLA-B*27-associated spondyloarthropathies, such as psoriatic arthritis, acute anterior uveitis, juvenile idiopathic arthritis and Crohn’s disease, as recent studies show universal representation of the characteristic TRBV9 CDR3 motif in HLA-B*27^+^ patients^6,7^.

In the future, such targeted elimination of the underlying cause of the disease, without systemic immunosuppression, could become applicable to some other autoimmune disorders, for which common disease-associated TCR motifs are being actively discovered, such as type 1 diabetes^16^, multiple sclerosis^21^ and non-HLA-B*27 associated Crohn’s disease^17^.

Furthermore, someday, a collection of therapeutic antibodies to a number of *TRBV* and *TRAV* gene segments will probably become available. This would make it possible to use an individualized immunotherapy approach based on the identification of clones involved in disease pathogenesis for each patient, and selection of an appropriate therapeutic antibody or a combination thereof.

## Methods

### Study approval

The study was approved by the ethics committee of Pirogov Russian National Research Medical University (protocol no. 221), and the patient provided written informed consent. The study was conducted according to CARE guidelines and in compliance with the principles of the Declaration of Helsinki.

The local ethics committee of the Research Institute of Medical Primatology approved animal experiments.

### Animal models

After quarantine, 12 *Macaca mulatta* males aged 5–8 years were selected for this experiment. For TCR repertoire profiling and real-time PCR monitoring, peripheral blood samples were collected immediately before and 3, 6, 14, 22, 40, 90, 150 and 300 days after a single i.v. administration of BCD-180 or human IgG immunoglobulins. We assigned each animal to one of three groups; two treatment groups and one control group (*n* = 4 animals per group). Animals in one treatment group received 1 mg BCD-180 i.v. and animals in the other received 10 mg of BCD-180 i.v. Animals in the control group received human i.v. IgG immunoglobulins (Microgen). Animals were randomized to the groups to ensure equal body weight per group. In a separate experiment, after quarantine, 40 *Macaca fascicularis* (20 males and 20 females) aged 4–7 years were each assigned to one of four groups (*n* = 10; 5 males and 5 females per group). Each group received either 3, 10 or 30 mg kg^−1^ of BCD-180 or placebo once every 2 weeks for 6 weeks, followed by a 20-week period without treatment. Animals were randomized to the groups to ensure equal body weight per group and sex. The animals were kept in accordance with the European Convention for the Protection of Vertebrate Animals used for Experimental and Other Scientific Purposes, with species-specific provisions for nonhuman primates.

### Immunotoxicity study in *Macaca fascicularis*

Peripheral blood was collected using the Vacuette blood system with heparin (Greiner Bio-One) immediately before and after BCD-180 injection at 3, 5, 7, 13, 18 and 25 weeks. The samples were prepared according to the manufacturer’s methodology (https://www.bdbiosciences.com/en-us/resources/protocols/stain-lyse-no-wash). Blood samples were incubated with the labeled antibody mix for 30 min at 37 °C and a humidity of 70–80%. Lysis of erythrocytes was performed using BD fluorescence-activated cell sorting (FACS) lysing solution (Becton Dickinson). The subpopulation composition of lymphocytes was assessed on a Guava easyCyte flow cytometer (Merck Millipore) using labeled antibody reagents CD3-PerCP-Cy5.5 clone SP34-2, CD4-FITC clone L200, CD8-PE clone RPA-T8, CD20-FITC clone 2H7, CD16-PE clone 3G8, and CD56-PE clone MY31 (BD Biosciences). This enabled us to quantify the following cell subsets: B cells (CD20^+^), T cells (CD3^+^), T_H_ cells (CD3^+^CD4^+^), cytotoxic CD8^+^ T cells (CD3^+^CD8^+^) and NK cells (CD3^−^CD16/56^+^). The data were processed using InCyte guavaSoft software. For analysis of the immunoglobulin composition, peripheral blood was collected using the Vacuette blood system with clot activator (Greiner Bio-One) to obtain at least 0.6 ml of serum immediately before and 6, 13 and 26 weeks after BCD-180 injection. The level of IgE in the serum was determined using the Monkey IgE ELISA kit (Life Diagnostics). IgA, IgG and IgM were detected in the blood serum on an HTI BioChem FC-360 automatic biochemical analyzer (High Technology), using standard kits for the detection of human immunoglobulins.

### TCRβ repertoire profiling

Peripheral blood samples were collected in Vacuette tubes with EDTA. PBMCs were isolated from 6 ml of peripheral blood by Ficoll (Paneco) density gradient centrifugation. PBMCs were washed with Hank’s buffer, and total RNA was extracted with the RNeasy Mini kit (Qiagen) with DNase treatment. Preparation of unique molecular identifier (UMI)-labeled TCRβ cDNA libraries was performed using the Monkey TCR RNA kit (MiLaboratories) and Human TCR RNA Multiplex kit (MiLaboratories) according to the manufacturer’s manuals. For cDNA synthesis, we used 200 ng of PBMC RNA. TRBV9-focused TCRβ cDNA libraries were prepared using an adapted version of the Human TCR RNA Multiplex kit (MiLaboratories). Sequencing was performed on an Illumina MiSeq and a NextSeq 550, using paired-end reads of 150 + 150 nucleotides. MIGEC^24^ was used for UMI-based read grouping and error correction. MiXCR^25^ was used for extraction of TCRβ CDR3 repertoires. VDJtools^26^ was used for downstream analyses.

### Quantification of TRBV9 abundance by real-time PCR

Peripheral blood samples from monkeys were collected immediately before and 21 days after the first injection of anti-TRBV9. Peripheral blood samples from the patient were regularly collected using the Vacuette blood collection system. PBMCs were isolated using the Ficoll (Paneco) gradient protocol, washed with Hank’s buffer, and then total RNA was extracted with the RNeasy Mini kit (Qiagen) including DNase treatment. cDNA synthesis was performed using SmartScribe reverse transcriptase (Takara Bio) according to the manufacturer’s protocol, with BC4short oligonucleotides (see Supplementary Table 1 for the oligonucleotides used). A total of 300 ng of RNA was used for first-strand cDNA synthesis. Real-time PCR reactions were performed in three replicates, using qPCRmix-HS SYBR (Evrogen) and the following specific oligonucleotides in a final concentration of 0.2 μM each: for *TRBV9*, forward *TRBV9*-specific and reverse BCuni2; for *TRBV7*, forward *TRBV7*-specific and reverse BCuni2; and for *TRBC*, forward BC_for_hum and reverse BC_rev_hum (only for patient samples). We next applied the 2^-ΔΔC^_T_ method to assess the difference in threshold cycle between targeted *TRBV9* and reference *TRBV7* gene segments, where *TRBV7* represents the sum of *TRBV7* gene segments, or between a *TRBV* and reference *TRBC* segment at the time-point before and at several time-points after anti-TRBV9 administration.

### Statistics

To test the equality of the medians of TRBV9/7 distributions between time-points, we used the Kruskal–Wallis test, followed by Dunnett’s test if necessary, with a 95% family-wise confidence level. Statistical analysis and visualization were performed using the R software environment v.3.14 (https://www.R-project.org) with the ggplot2 v3.4.3 (ref. ^27^) and ggseqlogo v0.1 (ref. ^28^) packages.

### Reporting summary

Further information on research design is available in the Nature Portfolio Reporting Summary linked to this article.

## Online content

Any methods, additional references, Nature Portfolio reporting summaries, source data, extended data, supplementary information, acknowledgements, peer review information; details of author contributions and competing interests; and statements of data and code availability are available at 10.1038/s41591-023-02613-z.

### Supplementary information

Supplementary Information Supplementary Note 1, Supplementary Fig. 1 and Supplementary Table 1.

Reporting Summary

### Source data

Source Data Fig. 2 Patient’s clinical data.

Source Data Extended Data Fig. 1 Real-time PCR data.

## Data Availability

TCRβ CDR3 repertoires and metadata are available in Figshare: https://figshare.com/projects/TRBV9_depletion_TCR_repertoires/171369. *Macaca mulatta* TCRβ CDR3 repertoires and metadata are available at 10.6084/m9.figshare.23609148.v2. Bulk TCR repertoires of the patient are available at 10.6084/m9.figshare.23609970.v3. TRBV9 TCR repertoires of the patient are available at 10.6084/m9.figshare.23611209.v2. Source data are provided with this paper.
