# Supplementary material for: Targeted depletion of TRBV9+ T cells as immunotherapy in a patient with ankylosing spondylitis
Source: Nat Med. 2023 Oct 23;29(11):2731–6. doi: 10.1038/s41591-023-02613-z (PMC10667094; doi:10.1038/s41591-023-02613-z)
Supplement: Supplementary file 1 — Supplementary Note 1, Supplementary Fig. 1 and Supplementary Table 1. [file 41591_2023_2613_MOESM1_ESM.pdf]

nature medicine

Brief Communication https://doi.org/10.1038/s41591-023-02613-z

Targeted depletion of TRBV9$^+$ T cells as immunotherapy in a patient with ankylosing spondylitis

In the format provided by the authors and unedited

Supplementary Note 1. Toxicity and local tolerance in preclinical studies.

To assess BCD-180 toxicity, we employed generally accepted experimental methods recommended for preclinical studies. These included monitoring of motor activity, vegetative reactions, the condition of the coat, changes in the eyes, the condition of the mucous membranes of the nose and oral cavity, stool, appetite, breathing pattern, and hematology parameters. We also assessed the effect of BCD-180 on blood coagulation; the protein, carbohydrate and enzymatic functions of the liver, including the activity of indicator enzymes, the level of total bilirubin, cholesterol, total protein, triglycerides, and glucose; function of the urinary system according to the results of clinical chemistry (level of urea, creatinine, sodium and potassium ions) and the results of urinalysis; the cardiovascular system according to ECG; and the central nervous system. Animals were subjected to euthanasia for pathomorphological examination of internal organs according to the standard program. No animal deaths were recorded during the study.

The clinical examination showed that the animals tolerated administration of BCD-180 well. During the entire period of observation, we observed no changes in autonomic reactions, the condition of the eyes, mucous membranes of the nose and mouth, or breathing patterns. We also saw no changes in the behavior of animals or perturbations of central nervous system function. For the entirety of the experiment, there was no significant change in body weight, nor any thermogenic reaction caused by BCD-180. During the clinical examination, we noticed the following minor clinical symptoms: dry skin, dandruff, and dullness of the coat. These changes were mild and reversible. Unstable stools were also noted in some animals. In two animals from the minimum and intermediate dose groups, slight exsicosis was noted at 12 weeks. The observed changes were mild and not systemic in nature.

We did not observe any effect on the number of red blood cells or hemoglobin concentration of the treated animals. Also, there were no meaningful changes in the numbers of eosinophils, neutrophils, lymphocytes, leukocytes, basophils, or platelets following BCD-180 administration. We observed sporadic changes in erythrocyte sedimentation rate and monocytes in individual animals, but taking into account the results of biochemical, immunological and pathomorphological studies, we considered these to be unrelated to the administration of BCD-180. In all groups of animals treated with the BCD-180, there was a decrease in TRBV9^{+} T lymphocytes compared with the control group. Depletion of the target population of T lymphocytes after intravenous administration of BCD-180 was equally pronounced in both females and males and persisted throughout the entire observation period.

According to histological studies, BCD-180 does not have a local irritant effect. Pathomorphological study revealed no adverse effect of BCD-180 on organs and organ systems, and the morphological structure of the internal organs in most cases corresponded to age and histological norms. Repeated administration of BCD-180 had no effect on the functional state of the liver (protein-synthetic function,

lipid and carbohydrate metabolism, activity of indicator enzymes), as well as the, urinary, cardiovascular, central nervous, respiratory, or hemostasis systems of experimental animals. Our data indicated that BCD-180 did not have adverse effects on the main organs and organ systems of *M. fascicularis* during repeated (once every two weeks) intravenous administration at doses of up to 30 mg/kg for 6 weeks.

Immunotoxicity

The dynamics of relative counts of T cells, T helper cells, cytotoxic T cells, B cells, and NK cells were similar for groups receiving the BCD-180 at doses of 3 mg/kg, 10 mg/kg and 30 mg/kg, and for the control group of animals. During the administration-free period, we saw no significant differences from the baseline in any of the groups. Thus, BCD-180 had no effect on the peripheral blood lymphocyte subsets of cynomolgus monkeys. The dynamics of the concentration of IgA, IgG, and IgM did not depend on the dose of BCD-180 and was comparable to the placebo group. When assessing the allergenic activity of BCD-180, we found that repeated intravenous administration of BCD-180 was not accompanied by an increase in IgE levels in the blood serum. We also saw no increase in the proliferative activity of lymphocytes associated with the introduction of BCD-180. These data indicate that BCD-180 did not have adverse effects on the immune system of *M. fascicularis*.

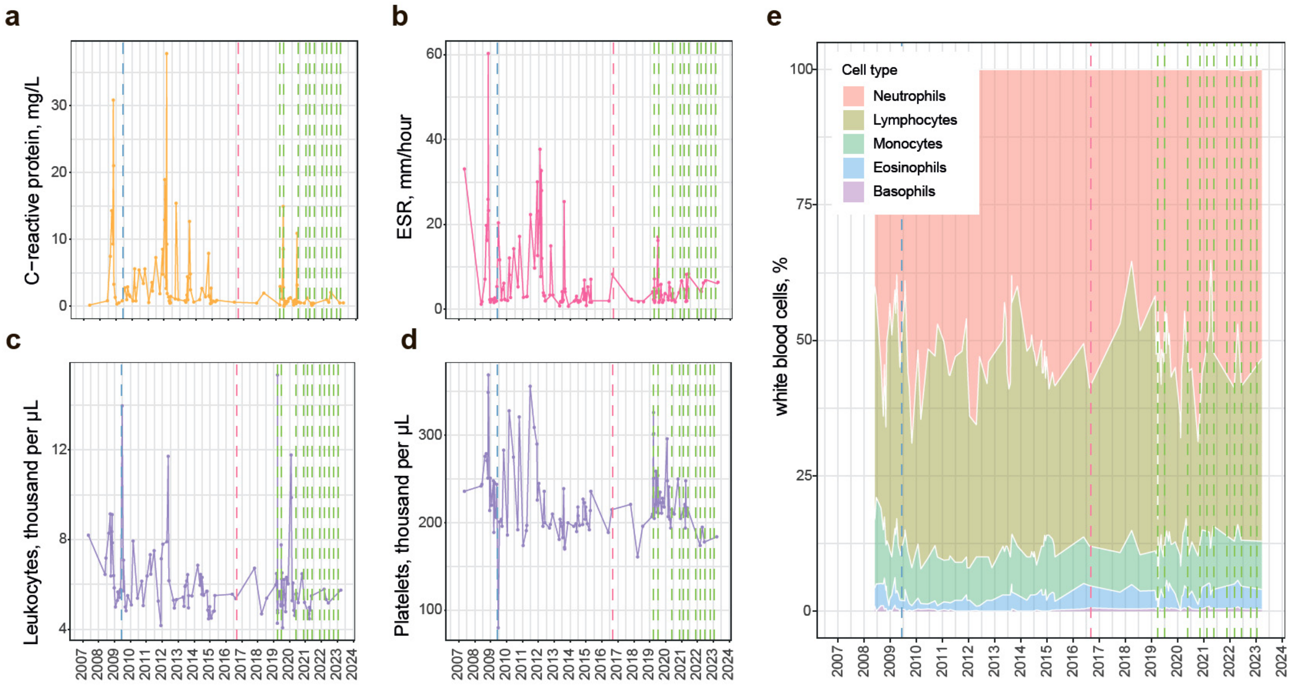

Supplementary Figure 1. Blood parameters. a. C-reactive protein (CRP), mg/L. **b**. Erythrocyte sedimentation rate (ESR), mm/hr. **c**. Leukocyte count, thousand per μL. **d**. Platelet count, thousand per μL. **e**. White blood cells content, %.

Supplementary Table 1. RT-PCR oligonucleotides.

Description	Oligonucleotide	Sequence 5'->3'
**Monkey RT-PCR system**		
cDNA synthesis	BC4short_mon	GTATCGGAGTCCTCGA
Forward PCR	TRBV9_uni	CTGACTTGCACTCTGAACTAAAC
Forward PCR	TRBV7_mon	TCTCCACTCTGACGATCCAGC
Reverse PCR	Rev_BC2Uni	TGCTTCTGATGGCTCAAACAC
**Human RT-PCR system**		
cDNA synthesis	BC4short_hum	GTATCTGGAGTCATTGA
Forward PCR	TRBV9_uni	CTGACTTGCACTCTGAACTAAAC
Forward PCR	TRBV7_hum	TCTCCACTCTGAAGATCCAGC
Reverse PCR	Rev_BC2Uni	TGCTTCTGATGGCTCAAACAC
Forward PCR	BC_for_hum	CTGTGTTTGAGCCATCAGAA
Reverse PCR	BC_rev_hum	GTCSGTGCTGACCCCACTG
