# Supplementary material for: Targeted depletion of TRBV9+ T cells as immunotherapy in a patient with ankylosing spondylitis
Source: Nat Med. 2023 Oct 23;29(11):2731–6. doi: 10.1038/s41591-023-02613-z (PMC10667094; doi:10.1038/s41591-023-02613-z)
Supplement: Supplementary file 2 — Reporting Summary [file 41591_2023_2613_MOESM2_ESM.pdf]

nature portfolio

Corresponding author(s): Dmitry Chudakov

Last updated by author(s): Sep 19, 2023

Reporting Summary

Nature Portfolio wishes to improve the reproducibility of the work that we publish. This form provides structure for consistency and transparency in reporting. For further information on Nature Portfolio policies, see our Editorial Policies and the Editorial Policy Checklist.

Statistics

For all statistical analyses, confirm that the following items are present in the figure legend, table legend, main text, or Methods section.

n/a	Confirmed	
☐	☒	The exact sample size (*n*) for each experimental group/condition, given as a discrete number and unit of measurement
☐	☒	A statement on whether measurements were taken from distinct samples or whether the same sample was measured repeatedly
☐	☒	The statistical test(s) used AND whether they are one- or two-sided<br>*Only common tests should be described solely by name; describe more complex techniques in the Methods section.*
☒	☐	A description of all covariates tested
☒	☐	A description of any assumptions or corrections, such as tests of normality and adjustment for multiple comparisons
☐	☒	A full description of the statistical parameters including central tendency (e.g. means) or other basic estimates (e.g. regression coefficient) AND variation (e.g. standard deviation) or associated estimates of uncertainty (e.g. confidence intervals)
☐	☒	For null hypothesis testing, the test statistic (e.g. *F*, *t*, *r*) with confidence intervals, effect sizes, degrees of freedom and *P* value noted<br>*Give P values as exact values whenever suitable.*
☒	☐	For Bayesian analysis, information on the choice of priors and Markov chain Monte Carlo settings
☒	☐	For hierarchical and complex designs, identification of the appropriate level for tests and full reporting of outcomes
☒	☐	Estimates of effect sizes (e.g. Cohen's *d*, Pearson's *r*), indicating how they were calculated

Our web collection on statistics for biologists contains articles on many of the points above.

Software and code

Policy information about availability of computer code

Data collection	No software used.
Data analysis	MIGEC v1.2.9 was used for UMI-based read grouping and error correction. MiXCR v3.0.13 was used for extraction of TCRβ CDR3 repertoires. VDJtools v1.2.1 was used for downstream post-analysis. Statistical analysis and visualization were performed using the R software environment (https://www.R-project.org/) and packages ggplot2 v3.4.3 and ggseqlogo v0.1.

For manuscripts utilizing custom algorithms or software that are central to the research but not yet described in published literature, software must be made available to editors and reviewers. We strongly encourage code deposition in a community repository (e.g. GitHub). See the Nature Portfolio guidelines for submitting code & software for further information.

Data

Policy information about availability of data

All manuscripts must include a data availability statement. This statement should provide the following information, where applicable:

- Accession codes, unique identifiers, or web links for publicly available datasets
- A description of any restrictions on data availability
- For clinical datasets or third party data, please ensure that the statement adheres to our policy

TCRβ CDR3 repertoires and metadata are available in Figshare Project: https://figshare.com/projects/TRBV9_depletion_TCR_repertoires/171369.
Macaca mulatta TCRβ CDR3 repertoires and metadata: https://doi.org/10.6084/m9.figshare.23609148.v2

Bulk TCR repertoires of the patient: https://doi.org/10.6084/m9.figshare.23609970.v3
TRBV9 TCR repertoires of the patient: https://doi.org/10.6084/m9.figshare.23611209.v2

Research involving human participants, their data, or biological material

Policy information about studies with human participants or human data. See also policy information about sex, gender (identity/presentation), and sexual orientation and race, ethnicity and racism.

Reporting on sex and gender	Male patient.
Reporting on race, ethnicity, or other socially relevant groupings	Caucasian patient.
Population characteristics	The patient, male, was born in 1963 from parents without chronic diseases and developed normally. Gender was determined based on self-report.
Recruitment	Recruited patient is a classic central HLA-B27-positive ankylosing spondylitis with long term history of therapy. Against the background of ongoing anti-TNF therapy, the patient continued to experience pain and stiffness in all parts of the spine, with severe limitation of movement in the cervical spine and pain in the hip joint.
Ethics oversight	The study was approved by the ethical committee of Pirogov Russian National Research Medical University (protocol №221).

Note that full information on the approval of the study protocol must also be provided in the manuscript.

Field-specific reporting

Please select the one below that is the best fit for your research. If you are not sure, read the appropriate sections before making your selection.

☒ Life sciences ☐ Behavioural & social sciences ☐ Ecological, evolutionary & environmental sciences

For a reference copy of the document with all sections, see nature.com/documents/nr-reporting-summary-flat.pdf

Life sciences study design

All studies must disclose on these points even when the disclosure is negative.

Sample size	Rationale for sample size chosen was determined by the expected prominence of TRBV9 T cells depletion, and ethical limitations that require minimization of animals used per study. 12 Macaca mulatta males aged 5–8 years were selected for the 1st experiment. For TCR repertoire profiling and RT-PCR monitoring, peripheral blood samples were collected before and 3, 6, 14, 22, 40, 90, 150, and 300 days after a single administration of BCD-180. We divided the animals into two groups (n = 4 for each group) that received 1 mg or 10 mg BCD-180 i.v. per animal, along with a control group that included four animals and received human intravenous IgG immunoglobulins. In a separate experiment, after quarantine, 40 Macaca fascicularis animals (20 males and 20 females) aged 4–7 years were enrolled. Four groups (n = 5 each of females and males per group) received 3, 10, or 30 mg/kg of BCD-180 or placebo once every two weeks for six weeks, followed by a 20-week period without treatment.
Data exclusions	No data exclusions.
Replication	TRBV9 depletion was performed in 2 independent experiements on 12 Macaca mulatta and 40 Macaca fascicularis animals. Both experiments were fully successful in respect of TRBV9 depletion.
Randomization	Animals were randomized to the groups to ensure equal body weight per group and sex.
Blinding	No blinding was performed since these experiments have well-defined primary outcome that is objective and not subject to interpretation or bias (TRBV9 depletion).

Reporting for specific materials, systems and methods

We require information from authors about some types of materials, experimental systems and methods used in many studies. Here, indicate whether each material, system or method listed is relevant to your study. If you are not sure if a list item applies to your research, read the appropriate section before selecting a response.

Materials & experimental systems

n/a	Involved in the study
☐	☒ Antibodies
☒	☐ Eukaryotic cell lines
☒	☐ Palaeontology and archaeology
☐	☒ Animals and other organisms
☒	☐ Clinical data
☒	☐ Dual use research of concern
☒	☐ Plants

Methods

n/a	Involved in the study
☒	☐ ChIP-seq
☐	☒ Flow cytometry
☒	☐ MRI-based neuroimaging

Antibodies

Antibodies used	The subpopulation composition of lymphocytes was assessed on a Guava® easyCyte flow cytometer (Merck Millipore, USA) using labeled antibody reagents manufactured by BD Biosciences, USA: CD3-PerCP-Cy5.5 lot: 9171962, Clone SP34-2, CD4-FITC lot: 6056755, Clone L200, CD8-PE lot: 7191542, Clone RPA-T8, CD20-FITC lot: 7235971, Clone 2H7, CD16- PE lot: 7130957, Clone 3G8, CD56-PE lot: 6246596, Clone MY31.
Validation	All antibodies were validated for Rhesus (Macaca mulatta) and Cynomolgus (Macaca fascicularis) by BD Biosciences, USA.

Animals and other research organisms

Policy information about studies involving animals; ARRIVE guidelines recommended for reporting animal research, and Sex and Gender in Research

Laboratory animals	12 Macaca mulatta aged 5–8 years and 40 Macaca fascicularis aged 4–7 years.
Wild animals	No.
Reporting on sex	12 Macaca mulatta males, 40 Macaca fascicularis (20 males and 20 females).
Field-collected samples	No.
Ethics oversight	Animals were kept in accordance with the guidelines for accommodation and care of laboratory animals, with species-specific provisions for nonhuman primates. The local ethical committee of Research Institute of Medical Primatology approved animal experiments.

Note that full information on the approval of the study protocol must also be provided in the manuscript.

Flow Cytometry

Plots

Confirm that:

☐ The axis labels state the marker and fluorochrome used (e.g. CD4-FITC).

☐ The axis scales are clearly visible. Include numbers along axes only for bottom left plot of group (a 'group' is an analysis of identical markers).

☐ All plots are contour plots with outliers or pseudocolor plots.

☐ A numerical value for number of cells or percentage (with statistics) is provided.

Methodology

Sample preparation	The samples were prepared according to the manufacturer's methodology (https://www.bdbiosciences.com/en-us/resources/protocols/stain-lyse-no-wash).
Instrument	Guava easyCyte flow cytometer (Merck Millipore, USA)
Software	The data were processed using InCyteTM guavaSoftTM software.
Cell population abundance	5000 events were collected for each sample.

Gating strategy

To determine blood lymphocyte subsets, a morphological gate was used on a two-parameter FSC vs SSC histogram. A population of cells in the unstained sample considered as a boundary for negative population. We do not report images of flow cytometry, only referencing these results in Supplementary Note 1.

☐ Tick this box to confirm that a figure exemplifying the gating strategy is provided in the Supplementary Information.
